# Futureproofing [^18^F]Fludeoxyglucose manufacture at an Academic Medical Center

**DOI:** 10.1186/s41181-018-0048-x

**Published:** 2018-10-05

**Authors:** Alexandra R Sowa, Isaac M Jackson, Timothy J Desmond, Jeremiah Alicea, Anthony J Mufarreh, Jonathan M Pham, Jenelle Stauff, Wade P Winton, Maria V Fawaz, Bradford D Henderson, Brian G Hockley, Virginia E Rogers, Robert A Koeppe, Peter J H Scott

**Affiliations:** 1 Department of Radiology, University of Michigan, Ann Arbor, MI 48109 USA; 2 Department of Medicinal Chemistry, University of Michigan, Ann Arbor, MI 48109 USA

**Keywords:** PET radiochemistry, Cyclotron targetry, Fludeoxyglucose (FDG), Fluorine-18, Automation

## Abstract

**Background:**

We recently upgraded our [^18^F]fludeoxyglucose (FDG) production capabilities with the goal of futureproofing our FDG clinical supply, expanding the number of batches of FDG we can manufacture each day, and improving patient throughput in our nuclear medicine clinic. In this paper we report upgrade of the synthesis modules to the GE FASTLab 2 platform (Phase 1) and cyclotron updates (Phase 2) from both practical and regulatory perspectives. We summarize our experience manufacturing FDG on the FASTLab 2 module with a high-yielding self-shielded niobium (Nb) fluorine-18 target.

**Results:**

Following installation of Nb targets for production of fluorine-18, a 55 μA beam for 22 min generated 1330 ± 153 mCi of [^18^F]fluoride. Using these cyclotron beam parameters in combination with the FASTLab 2, activity yields (AY) of FDG were 957 ± 102 mCi at EOS, corresponding to 72% non-corrected AY (*n* = 235). Our workflow, inventory management and regulatory compliance have been greatly simplified following the synthesis module and cyclotron upgrades, and patient wait times for FDG PET have been cut in half at our nuclear medicine clinic.

**Conclusions:**

The combination of FASTlab 2 and self-shielded Nb fluorine-18 targets have improved our yield of FDG, and enabled reliable and repeatable manufacture of the radiotracer for clinical use.

## Background

The global radiopharmaceuticals market is expected to reach $10 billion by 2024, driven by growing demand for diagnostic imaging procedures (positron emission tomography (PET) and single photon emission computed tomography (SPECT)) as well as the introduction of radiotherapeutics such as Lutathera® and Xofigo® and their approval by the U.S. Food and Drug Administration (FDA). In the context of PET imaging, [^18^F]fludeoxyglucose (FDG) remains the most widely utilized radiopharmaceutical in the world. The 1990s saw approval of FDG by the FDA and subsequent reimbursement by the Centers for Medicare and Medicaid Services (CMS), and since that time the use of FDG PET for imaging applications in oncology, neurology and cardiology has grown steadily. Reflecting this, an estimated 1.945 million clinical PET (and PET/CT) studies occurred in the United States in 2017, up 15% from 2015, and makes FDG PET a multimillion dollar market in its own right (PET Imaging Market Summary Report 2018).

The short half-life of fluorine-18 (109.77 min) necessitates that FDG is manufactured in a radiochemistry facility, either on site in an academic medical center or at a centralized nuclear pharmacy in close proximity to the clinical PET scanner(s). The daily synthesis and delivery of FDG for human studies presents unique challenges including: i) a need for rapid and reliable manufacturing since using short-lived ^18^F mandates a total preparation time of ~ 1–2 h and batch production on a daily basis; ii) radiation safety as multi-Curie amounts of ^18^F are often employed in the synthesis of FDG; iii) pharmaceutical-quality procedures to accommodate FDA regulated current Good Manufacturing Practice (cGMP); iv) reliability to ensure excellent patient care; and v) economic considerations to maintain cost-containment in health care operations. Initially, FDG was prepared using early homemade remote synthesis systems under pharmaceutical compounding regulations (for a review of FDG synthesis and quality control methods, see: Yu 2006). However, a changing regulatory environment in addition to the need for synthesizing larger amounts of FDG to meet the growth in FDG PET utilization have catalyzed a number of changes in the PET community from regulatory and practical points of view. For example, the requirement to manufacture FDG for clinical use as a generic drug in accordance with cGMP became a mandate in the United States in 2012. The cGMP regulations for PET drugs are described in 21CFR212 and enforced by the FDA (for an overview of PET drug regulatory considerations, see: Schwarz et al. 2014). Cassette-based automated synthesis modules have also become commonplace in FDG production sites, replacing the manual techniques and earlier homemade systems employed in the 1980s and 1990s (for a perspective on future technology developments as they pertain to PET radiochemistry, see: Thompson et al. 2016). Such systems facilitate compliance with cGMP as they can be run by software compliant with 21CFR11 (the FDA regulations covering electronic records) and utilize single use cassettes that are sterile, manufactured according to cGMP and which are compatible with cleaning validation protocols (Haka et al. 2017). In addition, the transition to automated systems has also enabled the production of larger amounts of FDG, sometimes in excess of > 20 Ci, in a manner that still ensures the safety of production radiochemists and adheres to the As Low As Reasonably Achievable (ALARA) principle.

**Fig. 1:**
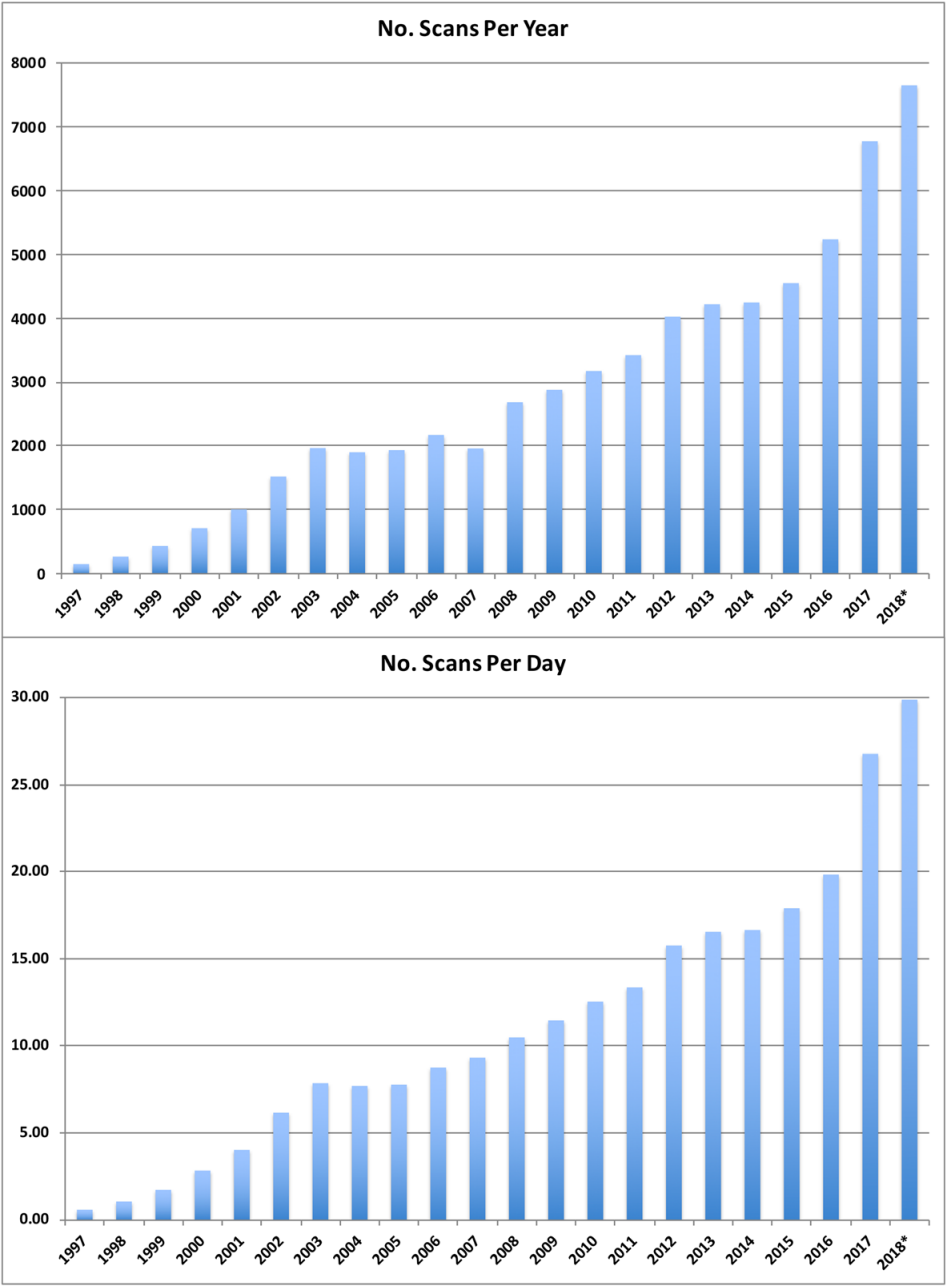
Clinical PET utilization at the University of Michigan PET Center (*estimated)

**Fig. 2:**
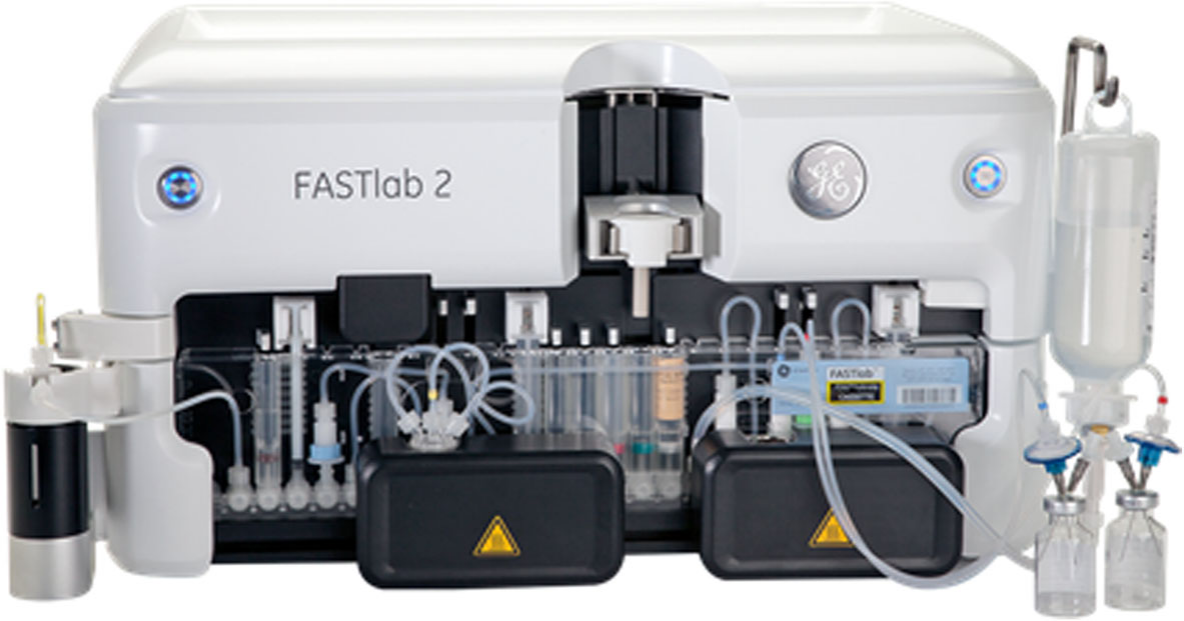
FASTLab 2 equipped with dual run FDG DUO cassette (image courtesy of GE Healthcare)

**Fig. 3:**
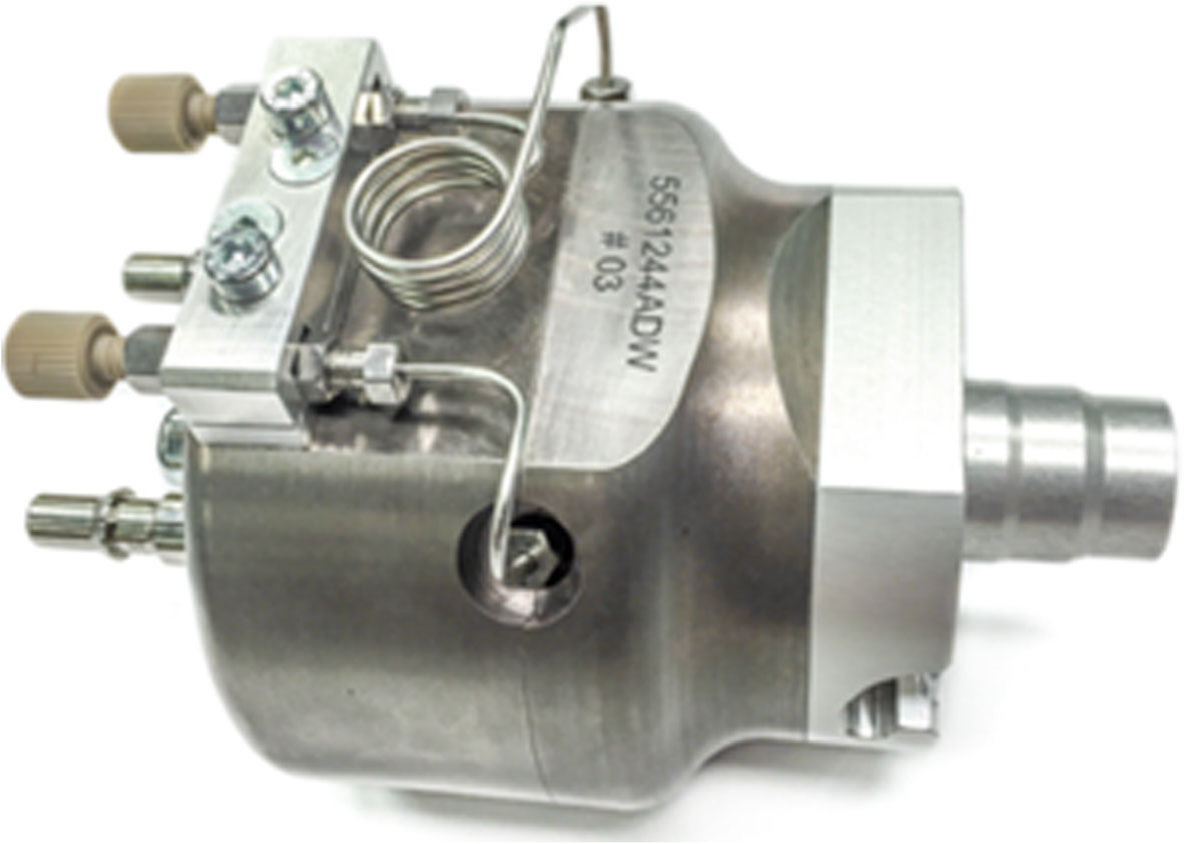
Self-shielded high yielding niobium target for production of fluorine-18 (image courtesy of GE Healthcare)

Since 2006, the University of Michigan PET Center has generated fluorine-18 on a General Electric (GE) PETTrace cyclotron equipped with silver (Ag) fluorine-18 targets, and manufactured FDG using the GE TRACERLab_MX-FDG_ synthesis module (formerly the FDG Synthesizer from Coincidence Technologies, until its acquisition by GE in 2001 (GE plans acquisition of PET device maker Coincidence Technologies 2001)). As of 2015, we have also manufactured generic FDG under an approved Abbreviated New Drug Application (ANDA). In the last two decades there has been steadily increasing demand for clinical PET within Michigan Medicine, the vast majority (> 80–90%) of which are FDG scans (Fig. 1). For example, 151 patients received PET scans in 1997 (0.6 per day over 250 scanning days), compared to 1960 in 2007 (9.3 per day over 210 scanning days), and 6777 in 2017 (26.79 per day over 253 scanning days). In addition, we have received a number of inquiries about the possibility of our center supplying FDG to various outside entities which we are actively exploring. Coupled with an “end-of-life” announcement from GE for the TRACERLab_MX-FDG_, we had an urgent need to futureproof our FDG production capabilities, while also increasing FDG yields and expanding the number of batches of FDG we could manufacture each day. In order to address these issues, we have updated our FDG manufacturing program in two phases. Phase 1 consisted of replacing TRACERLab_MX-FDG_ single cassette synthesis modules with FASTLab 2 modules and DUO cassettes (Fig. 2), while Phase 2 involved updates to the cyclotron, including upgrading the fluorine-18 targets on the PETTrace from traditional Ag targets to high yielding self-shielded Nb targets (Fig. 3). In this article we report our experience with these upgrades from both practical and regulatory perspectives, and summarize our experience manufacturing FDG at each phase.

## Methods

### General considerations

Unless otherwise stated, kits and supplies were commercially available and used as received: TRACERLab_MX-FDG_ cassettes were purchased from ABX or Rotem, FASTLab 2 DUO cassettes were purchased from GE, and Modular Lab dispensing cassettes were obtained from Eckert and Ziegler. H_2_^18^O was obtained from ABX or Rotem. Sodium phosphates, USP and sterile water for injection, USP were purchased from Hospira. Sterile product vials were purchased from Hollister-Stier.

### Generation of [^18^F]fluoride on the GE PETtrace cyclotron

[^18^F]Fluoride was produced via the ^18^O(p,n)^18^F nuclear reaction using a 16 MeV GE PETTrace cyclotron using either Ag or Nb targets as summarized in Table 1.

**Table 1:** Production of [^18^F]fluoride on a PETtrace Cyclotron

	Beam parameters	Target	Volume of H_2_^18^O	Starting fluoride (mCi)
Historical (*n* = 2137)	40 μA, 22 min	Ag	1.6 mL	1011 ± 116
Phase 1 (*n* = 383)	40 μA, 30 min	Ag	1.6 mL	1179 ± 106
Phase 2 (n = 235)	55 μA, 22 min	Nb	2.7 mL	1330 ± 153

### Synthesis of [^18^F]Fludeoxyglucose

**Scheme 1:**
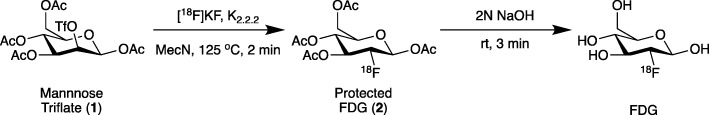
Synthesis of FDG

**Fig. 4:**
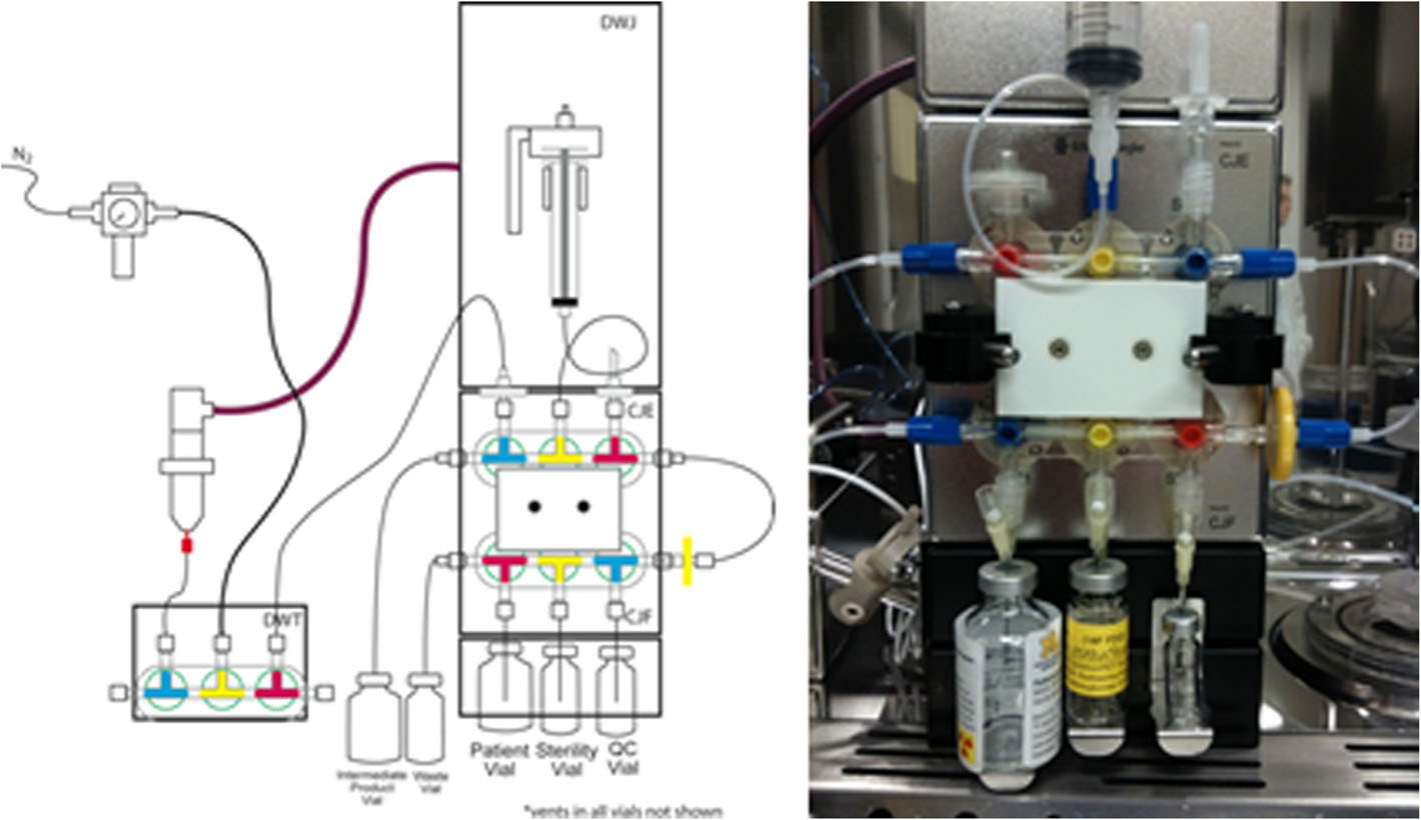
Modular Lab Automated Dispensing System

The synthesis of FDG was performed on either a GE TRACERLab_MX-FDG_ as previously described (Richards and Scott 2012), or FASTLab 2 synthesis module as follows: [^18^F]fluoride was delivered from the cyclotron and trapped on a quarternary methylammonium (QMA) cartridge. The [^18^F]fluoride was eluted from the QMA into the reactor with an eluent mixture (0.5 mL of a solution containing 3.75–5.75 mg of K_2_CO_3_ and 23–30 mg of kryptofix-2.2.2 (K_2.2.2_) in a 1:4 mixture of water and acetonitrile) and azeotropically dried. A solution of mannose triflate **1** (33 ± 3 mg in 1.7 mL acetonitrile) was added and the radiofluorination was conducted at 125 °C for 2 min to give [^18^F]fluoro-1,3,4,6-tetra-*O*-acetyl-D-glucose ([^18^F]FTAG, **2**) (Scheme 1). The reaction mixture containing **2** was diluted with water (11 mL) and passed through a C-18 Sep-Pak cartridge. [^18^F]FTAG (**2**) was trapped on the C-18 Sep-Pak cartridge, washed with additional water (13 mL), and base hydrolysis occurred on the cartridge at room temperature using 2 N NaOH (1 mL) for 3 min to give FDG. FDG was then eluted from the cartridge with citrate buffer (1.7 mL), passed through a second C-18 Sep-Pak (to remove any partially-protected FDG) followed by an alumina-N cartridge (to remove any unreacted [^18^F]fluoride ion) and diluted with water (~ 19 mL) before being dispensed into a sterile intermediate vial pre-charged with water for injection, USP (7.6 mL), and sodium phosphate buffer for injection, USP (0.35 mL). The resulting solution (~ 29 mL) was then passed through a 0.22 μm sterile filter, and dispensed into patient (~ 26 mL), quality control (~ 0.5 mL) and sterility (~ 2.5 mL) vials using an Eckert and Ziegler Modular Lab automated dispensing system (Fig. 4).

### Quality control of [^18^F]Fludeoxyglucose

Quality control (QC) testing of FDG doses was conducted according to the guidelines outlined in 21CFR212, and as previously described (Richards and Scott 2012). Daily QC testing consisted of visual inspection (doses must be clear, colorless and free of particulates), pH (pH paper, must be 4.5–7.5), residual K_2.2.2_ (spot test, must be ≤50 μg/mL), radiochemical purity (TLC, must be > 90%), radiochemical identity (TLC, R_F_ of radiotracer and reference standard match), radionuclidic identity (half-life must be 105–115 min), residual solvent analysis (GC, < 410 ppm MeCN; < 5000 ppm EtOH), sterile filter integrity (bubble point, ≥50 psi), bacterial endotoxin analysis (Endosafe, ≤175 endotoxin units / dose), and sterility per USP Chapter 71 (fluid thioglycolate media and soybean casein digest agar media tubes, no evidence of microbial growth found). Additional periodic QC testing including radionuclidic purity (MCA, ≥99.5%) and osmolality (osmometer, 270–330 mOsmol/kg) was conducted quarterly. All doses of FDG discussed in this article met or exceeded all of these quality control release criteria, and were stable for 24 h after end-of-synthesis (EOS).

## Results and discussion

### Practical considerations

Historically, we have generated fluorine-18 on a GE PETTrace cyclotron equipped with Ag fluorine-18 targets, and manufactured FDG using the GE TRACERLab_MX-FDG_ synthesis module (Table 2). A typical production of FDG began by generating [^18^F]fluoride with the PETtrace cyclotron via the ^18^O(p,n)^18^F reaction. Bombarding an Ag target containing H_2_^18^O (~ 1.6 mL) with a 40 μA proton beam for 22 min produced 1011 ± 116 mCi of [^18^F]fluoride. FDG was then prepared by standard fluorination of mannose triflate (**1**), followed by base hydrolysis (Scheme 1). Typical non-corrected yields of FDG using this setup were 527 ± 95 mCi (*n* = 2137), corresponding to 52% activity yield (AY). This amount of FDG was adequate when scanning up to ~ 15 or 16 patients per day at Michigan Medicine. However, as discussed above, there has been steadily increasing demand for FDG within our PET Center (Fig. 1), as well as inquiries about the possibility of our center supplying FDG to a number of outside entities. Concurrent with this growth, GE released an “end-of-life” announcement for the TRACERLab_MX-FDG_ because of obsolete parts. As a result, in Phase 1 of the updates to our FDG production operation, two TRACERLab_MX-FDG_ modules were replaced with FASTLab 2 synthesis modules.

**Table 2:** FDG Production Data

	Starting ^18^F^−^ (mCi)	Synthesis Module	FDG (mCi) ^a^	RCY^a^
Historical^b^ (*n* = 2137)	1011 ± 116	TRACERLab_MX-FDG_	527 ± 95	52%
Phase 1^b^ (*n* = 383)	1179 ± 106	FASTLab 2	839 ± 77	71%
Phase 2^b^ (n = 235)	1330 ± 153	FASTLab 2	957 ± 102	72%

a non-corrected yields at end-of-synthesis; b Historical: TRACERLab/Ag targets; Ph 1: FASTLab/Ag targets; Ph 2: FASTLab/Nb targets

From a practical perspective, the transition from TRACERLab_MX-FDG_ modules to FASTLab 2 was seamless. Replacement of both modules was completed within 7 business days, including Install Qualification and Operation Qualification (IQOQ). Since the DUO cassettes allow manufacture of two batches of FDG within a 26 h period, our production capacity increased to 200% of our previous levels; FDG production capacity of our lab doubled from 2 batches of FDG per day on 2 x TRACERLab_MX-FDG_ modules to 4 batches of FDG per day on 2 x FASTLabs. Notably, this increased capacity was accomplished without the need to install costly new hot-cells in the laboratory. We considered installing both FASTLabs in a single mini-cell since their size allows this configuration. This would have made one of our two mini-cells occupied by the TRACERLab_MX-FDG_ modules available for other applications. However, in the end we reasoned that having both FASTLabs in the same mini-cell would not allow us to run one while conducting maintenance on the other. Therefore we elected to install them in separate mini-cells. The transition to FASTLab also simplified our workflow and inventory management. Since all of the reagents for 2 FDG production runs are contained within a single FASTLab 2 cassette, manufacturing 2 batches of FDG per day is quite straightforward when compared to ordering, receiving and utilizing the separate hardware kits, reagent vials and other components required for 2 separate runs a day using the TRACERLab_MX-FDG_ modules.

In anticipation of an increase to ~ 25 patients scanned with FDG per day at our facility, we increased the beam time to 30 min and deployed the FASTLab 2 modules (Table 2). Thus, bombarding an Ag target containing H_2_^18^O (~ 1.6 mL) with a 40 μA proton beam for 30 min produced 1179 ± 106 mCi of [^18^F]fluoride. FDG was then produced using the FASTLab 2, and typical yields were 839 ± 77 mCi (*n* = 383), corresponding to 71% non-corrected AY. We were gratified to observe that the optimized manufacturing process developed for the FASTLab 2 offered a significant increase in AY of FDG (cf. 52% for the TRACERLab_MX-FDG_).

**Fig. 5:**
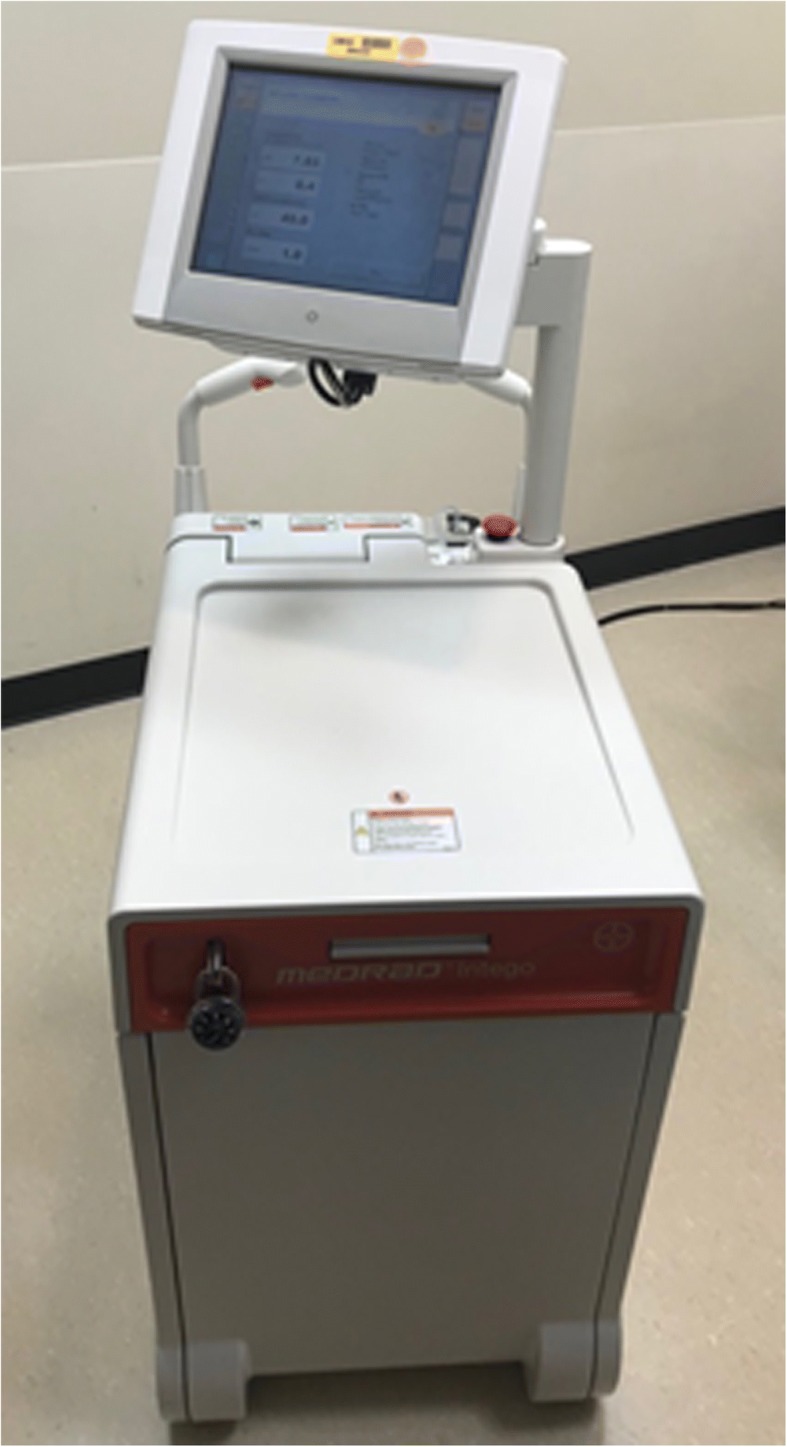
Bayer Medrad® Intego PET Infusion System

In late 2017, there was a move to further expand the number of patients that could be scanned with FDG to 30–32 per day. Operations at our PET Center are complex and include 4 radiotracers manufactured for routine clinical use, 35 different PET radiotracers manufactured routinely for clinical research, as well as a basic science research program developing new radiochemistry methodology and novel radiotracers. Given this operational complexity, we do not have bandwidth to monopolize the cyclotron for hours every day and run long fluorine-18 beams to increase our FDG output like commercial nuclear pharmacies. Moreover, in the clinic, multi-dose vials of FDG are used in combination with the Bayer Medrad® Intego PET Infusion System (Fig. 5). The Intego is compatible with vials containing 700–750 mCi of FDG. Above these radioactivity levels, dose preparation and radiation shielding are compromised (Bayer Medrad Intego Brochure 2017). We therefore wished to increase our yields of [^18^F]fluoride from the cyclotron (and the corresponding yields of FDG) within the confines of a short beam (≤30 min) such that an FDG beam could be efficiently slotted in around 10–12 other beams that are run on our PETTrace during a typical work day.

Phase 2 of our facility updates occurred in January 2018, when we undertook the manufacturer-recommended ten year life extension and refurbishment (TYLER) maintenance on the PETTrace cyclotron. This program was undertaken to update the cyclotron with the latest technology (water pump, magnet power supply etc.), while concurrently increasing our radionuclide production capacity. Updates included replacing outdated Ag body fluorine-18 targets with new high yield Nb body self-shielded fluorine targets. These targets use 2.7 mL of H_2_^18^O, and can be run on our updated system at beam currents up to 85 μA on a single target, or 130 μA on dual targets. As a side note, the tungsten-copper alloy in the self-shielded target is specified to lead to a 10 to 20-fold reduction in exposure resulting from the target foil, and 100-fold reduction in exposure due to any residual ^18^F in the target. We have only been working with the targets for 6 months but our initial experiences appear to be in line with these manufacturer specifications.

Following these system updates, our goal was to generate ~ 950 mCi of FDG at EOS. After removal of sterility and QC samples, this would provide ~ 850 mCi in the patient vial to be transported to the PET imaging suite. Allotting a further 0.5 h for radioactive decay associated with completion of QC testing and transporting FDG to the PET suite would allow us to deliver ~ 700 mCi, which is compatible with the limits of the Intego PET Infusion system (vide supra). After several optimization studies with the updated cyclotron, we decided upon a 55 μA proton beam for 22 min, which generates 1330 ± 153 mCi of [^18^F]fluoride (*n* = 235). Using these cyclotron beam parameters in combination with the DUO cassettes on FASTLab 2, yields of FDG were 957 ± 102 mCi at EOS, corresponding to 72% AY (Table 2). Manufacturing this amount of FDG at 8:00 am and 2:00 pm allows us to run two clinical PET-CT scanners for 12 h per day, and scan up to 32 patients a day with FDG.

### Regulatory considerations

When updating the manufacture of FDG, as an FDA-approved drug there were two main regulatory considerations to address in addition to the practical aspects outlined above. The first of these was establishing that FDG manufactured on the FASTLab (using Ag or Nb targets) was equivalent to FDG made using the TRACERLab_MX-FDG_, and that in each case the product matched the Reference Listed Drug (RLD) that was the basis for our ANDA (Table 3). An RLD is the approved drug product to which a new generic version is compared. The FDA Guidance on the content for PET drug ANDAs requires drug producers to demonstrate that the generic PET drug can be effectively substituted and provide the same benefit as the RLD drug that it copies (FDA Guidance 2011). The ANDA must show that the generic drug is the same as the RLD version in the following ways:

- The active ingredient in the generic drug is the same as in the RLD.
- The generic drug has the same strength, use indications, form (such as a tablet or an injectable), and route of administration.
- The inactive ingredients of the generic drug are acceptable and within +/− 5% of the RLD, or where such ingredients and their amounts have been previously approved in a drug product and do not significantly affect the physical or chemical properties of the drug.
- The generic drug is manufactured under the same standards as the RLD.
- The container in which the PET drug will be shipped and sold is appropriate, and the label is the same as the RLD label.

**Table 3:** Comparison of FDG with Reference Listed Drug after each update

	RLD Requirement	Historical^a^	Phase 1^a^	Phase 2^a^
Conditions of Use	Neurology, oncology, cardiology	Neurology, oncology, cardiology	Neurology, oncology, cardiology	Neurology, oncology, cardiology
Active Ingredient	FDG	FDG	FDG	FDG
Route of Administration	Intravenous	Intravenous	Intravenous	Intravenous
Dosage Form	Injection	Injection	Injection	Injection
Strength	20–300 mCi/mL (@ EOS)	24	29	33
Specific activity	No-carrier-added (NCA)	NCA	NCA	NCA
Inactive Ingredients	4.5 mg/mL NaCl in citrate buffer	4.58 mg/mL	4.55 mg/mL	4.55 mg/mL
Osmolality	Isotonic	Isotonic	Isotonic	Isotonic

^a^Historical: TRACERLab/Ag targets; Ph 1: FASTLab/Ag targets; Ph 2: FASTLab/Nb targets

Updating the manufacturing process had no impact on the active ingredient, dosage form, route of administration or conditions of use.

The strength (mCi/mL) of the product changed as a result of the increased yields, but this was a small change that was within the approved range for the RLD. Similarly, the NaCl concentration is slightly different for FDG produced on the FASTLab, stemming from a different product volume (29 mL for the FASTLab vs 22.25 mL for the TRACERLab), but the final NaCl concentration is within the range required (4.5 mg/mL ± 5%) to demonstrate that the generic drug is both qualitatively (Q1) and quantitatively (Q2) the same as the RLD and thus qualify for a waiver from full pharmacokinetic bioequivalence studies. Notably, in order to achieve this NaCl level, we need to further dilute the product obtained from both synthesis modules. Citrate buffer for injection, USP is not commercially available, diluting with 0.9% saline for injection, USP would change the NaCl concentration outside of the range of the RLD, and diluting with sterile water for injection, USP (SWFI) would alter the tonicity of the final product. For these reasons, we elected to dilute the product from the synthesis module with a mixture of commercially available SWFI and sodium phosphates for injection, USP (see Methods section) to achieve 4.5 mg/mL NaCl and an isotonic solution. These diluents are common components of other FDA-approved injectable drugs and, reflecting this, the formulation was granted a bioequivalence waiver and approved by FDA in our original ANDA submission.

The second major regulatory consideration was how to document changes to our manufacturing process with the FDA. Our original ANDA included use of both Ag and Nb targets to produce fluorine-18 and therefore did not represent a change to our process from a regulatory point of view. In contrast, our original ANDA only contained production of FDG using a TRACERLab_MX-FDG_. Pursuant to section VII.D.1 of the *FDA Guidance for Industry: Changes to Approved NDA or ANDA,* for drug products changes to equipment of the same design and operating principal is considered a minor change that can be documented in the next annual report submitted for the ANDA (FDA Guidance for Industry 2004). The FASTLab 2 has the same design and operating principal as the TRACERLab_MX-FDG_ identified in our original ANDA submission, and both systems are from the same manufacturer. Moreover, no additional changes were made to the manufacturing process, quality control, formulation specifications, aseptic processing, sterilization of the final product or labeling and, as stated above, consistency with the RLD was maintained. As such, we completed verification batches during Performance Qualification (PQ) of the FASTLab 2 modules prior to beginning clinical delivery of FDG to establish that product made using the FASTLab 2 met or exceeded all established quality specifications and was identical to that prepared on the TRACERLab_MX-FDG_ module. We documented the equipment change in our 2017 annual report, presenting a side-by-side comparison of the verification data from both synthesis modules to the FDA, and have been manufacturing FDG under our ANDA using FASTLab 2 for about 18 months.

## Conclusion

In summary, we have found the combination of FASTLab 2 and self-shielded Nb fluorine-18 targets enables reliable and repeatable manufacture of FDG. In the 18 months since we began using FASTLab 2 to manufacture FDG, it has proven to be a robust and reliable platform with an uptime > 99%. Our workflow, inventory management and regulatory compliance have been greatly simplified following the synthesis module and cyclotron upgrades and, as a result of our increased FDG production capacity, patient wait times for FDG PET have been cut in half from four days to two days at our nuclear medicine clinic.

## Abbreviations

ALARA: As low as reasonably achievable
AY: Activity yield
EOS: End-of-synthesis
EtOH: Ethanol
FDG: Fludeoxyglucose
K_2.2.2_: Kryptofix-2.2.2
MCA: Multichannel analyzer
MeCN: Acetonitrile
NCA: No-carrier-added
PET: Positron emission tomography
QC: Quality control
QMA: Quaternary methylammonium
SPECT: Single photon emission computed tomography
